# Point Projection Mapping System for Tracking, Registering, Labeling, and Validating Optical Tissue Measurements

**DOI:** 10.3390/jimaging10020037

**Published:** 2024-01-30

**Authors:** Lianne Feenstra, Stefan D. van der Stel, Marcos Da Silva Guimaraes, Behdad Dashtbozorg, Theo J. M. Ruers

**Affiliations:** 1 Image-Guided Surgery, Department of Surgical Oncology, Netherlands Cancer Institute, Plesmanlaan 121, 1066 CX Amsterdam, The Netherlands; s.vd.stel@nki.nl (S.D.v.d.S.); b.dasht.bozorg@nki.nl (B.D.); t.ruers@nki.nl (T.J.M.R.); 2 Department of Nanobiophysics, Faculty of Science and Technology, University of Twente, Drienerlolaan 5, 7522 NB Enschede, The Netherlands; 3 Department of Pathology, Netherlands Cancer Institute, Plesmanlaan 121, 1066 CX Amsterdam, The Netherlands; m.d.silva@nki.nl

**Keywords:** optical tissue sensing validation, Projection Mapping, histology correlation

## Abstract

The validation of newly developed optical tissue-sensing techniques for tumor detection during cancer surgery requires an accurate correlation with the histological results. Additionally, such an accurate correlation facilitates precise data labeling for developing high-performance machine learning tissue-classification models. In this paper, a newly developed Point Projection Mapping system will be introduced, which allows non-destructive tracking of the measurement locations on tissue specimens. Additionally, a framework for accurate registration, validation, and labeling with the histopathology results is proposed and validated on a case study. The proposed framework provides a more-robust and accurate method for the tracking and validation of optical tissue-sensing techniques, which saves time and resources compared to the available conventional techniques.

## 1. Introduction

Surgery combined with (neo)adjuvant therapy is currently the most-common treatment for patients with cancer. Oncological surgery is characterized by a delicate balance between radical tumor resection and sparing healthy tissue as much as possible. For a surgeon, recognizing tumor margins can be challenging since the resection of the tumor is mostly based on visual and tactile feedback. This can result in resections too close to the tumor (positive resection margins) or resections too far from the tumor, leading to the increased risk of tumor recurrence, undesired cosmetic outcomes, or potential damage to vital anatomical structures. Tumor-positive resection margins vary from 4.3% in uterine cancer to 35% in ovarian cancers, up to 19% in advanced rectal cancer [1], and 21% for prostate cancer [2]. In this case, additional treatment such as chemotherapy, radiotherapy, or surgical re-excision may be necessary, which affects the morbidity, as well as the quality of life of the patients [3]. In contrast, in breast cancer, the excised tissue volume of the resection specimen often exceeds 2–3-times the volume of the tumor, leading to worse cosmetic results [4,5]. Therefore, there is a need for more-precise oncological surgery, making it possible to detect tumor regions intraoperatively and, thereby, lowering the number of positive resection margins and additional treatments.

Optical technologies have shown great potential for the assessment of resection margins since they can reflect the biochemical and functional properties of the measured tissue. These technologies already have been successfully evaluated in multiple oncology domains for discriminating tumor from healthy tissue with high accuracies [6,7,8,9]. This includes Point-based measurement techniques such as Diffuse Reflectance Spectroscopy (DRS) [10,11], Raman Spectroscopy [12], Fluorescence Lifetime Imaging (FLIm) [13], and infrared Spectroscopy [14], as well as image-based techniques including hyperspectral imaging [15,16]. Optical tissue-sensing technologies have clinical advantages since they are non-destructive, and they do not require exogenous contrast with dyes. Besides, they have the potential to be performed in real-time, providing immediate feedback to the user.

**Figure 1:**
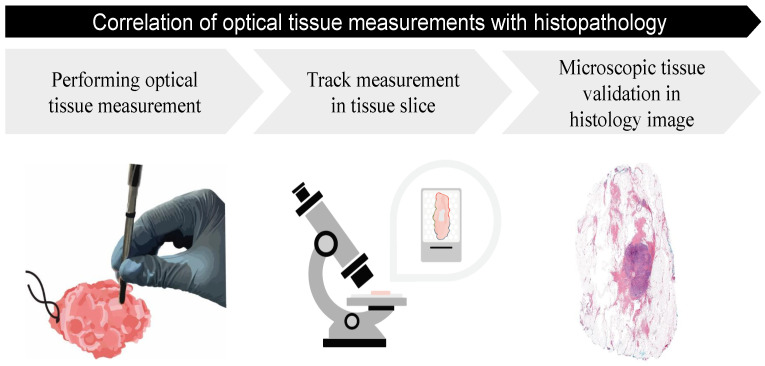
Ground truth validation of Point-based optical measurements: after the performed optical measurement, a tracking method is needed to trace the performed measurement area back in a gross-sectioned tissue slice. The gross-sectioned tissue slice will be further processed and result in a histology image (H&E tissue section). From this image, the optically measured tissue area can be defined microscopically and will be considered as the ground truth.

The first steps after the development of an optical tool involve ex vivo tissue specimen studies, where the technology will be evaluated for clinical purposes. In order to use optical technologies as a diagnostic tool for the optimization of surgical outcomes eventually, it is important that the optical tissue measurements are validated with a ground truth first [17]. Ground truth validation of optical tissue-sensing technologies is currently provided by hematoxylin-and-eosin (H&E)-stained tissue sections from which the measured tissue structures can be identified microscopically [18]. From this H&E section, a pathologist annotates all the different tissue structures located in the measured tissue area, which will then be considered as the ground truth. Accordingly, it is required to track where exactly on the excised tissue specimen the Point-based optical tissue measurements were performed in order to correlate those measurement locations in the gross-sectioned tissue slices and corresponding H&E-section annotations (Figure 1). The accurate correlation of an optical tissue measurement to histopathology is especially of importance for the development of (real-time) tissue-classification algorithms since the incorrect labeling of the data will influence the performance during the training of machine learning models. This correlation involves, for example, a registration between a microscopic histology image and a corresponding snapshot image of a tissue specimen.

For the development of accurate tissue classification algorithms and the validation of optical tissue-sensing techniques, an important first step consists of tracking the Point-based optical measurements performed on tissue specimens. Moreover, it has been observed that some studies have not had an adequate tracking method or they rely on visual correspondence only [19,20,21,22]. As a result, the correlation with histopathology is based on visual memory and, therefore, prone to human error. Other studies have shown conventional approaches to track the position of the optical tissue measurement, which involve the placement of ink marks or fiducial markers on the tissue specimen’s surface after acquisition [16,23] and the use of measurement grids and live-tracking of the optical probe [24,25]. However, these methods are limited since the accuracy of tracking can be affected by human errors and the placement of such markers can damage the tissue, complicating histopathology processing and analysis. For these reasons, it would be desirable to have a more-precise and -generalized method, applicable to the various optical tissue-sensing techniques available, which tracks optical tissue measurements in any desired location without damaging or marking the tissue specimens.

The second step should deal with the challenge of establishing a robust correlation between the tracked optical tissue measurement locations and the corresponding histopathological tissue labels. Establishing an accurate correlation between optical tissue measurements and the ground truth is especially of importance when preparing datasets for training supervised machine learning techniques for tissue discrimination. Using accurately labeled data, tissue classification algorithms can be developed to eventually classify tissue structures in real-time. The labeling of optical data often includes a multistep registration method, where, for example, a microscopic H&E section, including tissue annotations from a pathologist (ground truth), is registered to a white light specimen image [26,27,28]. With this registration, each tracked measurement can be labeled with the definite measured tissue type or tissue type percentages. However, due to histopathology processing, such as formalin fixation and paraffin embedding processes, the H&E sections are generally deformed compared to the optically measured tissue. These deformations include shrinkage, stretching, and compression of the microscopic tissue slices. Sometimes, tears and even the loss of tissue can be observed as a result of the slicing and staining process. So, simply overlaying images or using affine registration methods between the specimen images and microscopic H&E sections will be imprecise. Previous studies have shown the importance of accounting for tissue deformations when correlating optical tissue measurements with histological results [29]. Thus, when taking tissue deformations into account, an improvement in the correlation of optical tissue measurements could be achieved.

In this work, a new framework for accurate validation of Point-based optical tissue measurements will be introduced. The first part of this article focuses on the development of a Point Projection Mapping (PPM) pipeline in which we used a custom-built setup and also an off-the-shelf device. With each of these systems, it becomes possible to track and project any number of desirable measurement locations on the tissue specimen without damaging or marking the tissue and to work both with optical measurements performed on the surface of tissue specimens, as well gross-sectioned tissue slices. Consequently, a generalized method for tracking and registering Point-based optical tissue measurements to histopathology will be proposed. With improved labeling of optical measurements, more-accurate tissue classification algorithms can be developed and more-precise tissue discrimination during surgical procedures can be achieved. Besides, with an increased number of accurately labeled measurement locations, time and resource use can be decreased since a decreased number of specimens will be required to develop these classification algorithms. This presented approach is applicable to multiple specimen types and Point-based optical tissue-sensing techniques available.

The novel contributions of this paper can be summarized as follows:

- Developing a Point Projection Mapping (PPM) system, which allows for tracking of Point-based optical measurements performed on tissue specimens for the validation of optical tissue-sensing technologies.
- Introducing a newly developed framework for the registration, validation, and labeling of optical data with histopathology.
- Validating the proposed framework on a use-case scenario, namely Point-based optical tissue measurements performed on breast cancer lumpectomy specimens.

The remainder of this paper is organized as follows: Section 2.1 describes the development and technical information regarding the PPM setups. The proposed framework for the validation of optical tissue-sensing technologies will be presented in Section 2.2. The results of a use-case scenario are presented in Section 3, which is followed by the discussion and conclusion in Section 4 and Section 5, respectively.

## 2. Material and Methods

In this section, the developed Point Projection Mapping (PPM) setup is introduced first. Afterward, the proposed framework for the accurate correlation between optical tissue measurements with the histopathology results will be described, by using the PPM setup in a use-case study.

### 2.1. Point Projection Mapping

For this study, a PPM pipeline was developed, which allows the tracking of Point-based optical measurement locations for the validation of optical tissue-sensing technologies. With such a system, it is possible to project any number of desirable measurement locations on the tissue specimen without damaging or marking the tissue. Optical tissue measurements can be performed on each Point Projection separately and, later, be traced back in the histology images.

#### 2.1.1. Hardware

We employed two different setups for the PPM system: (1) a custom-built system and (2) the all-in-one HP Sprout Pro.

##### Custom-Built Setup

**Figure 2:**
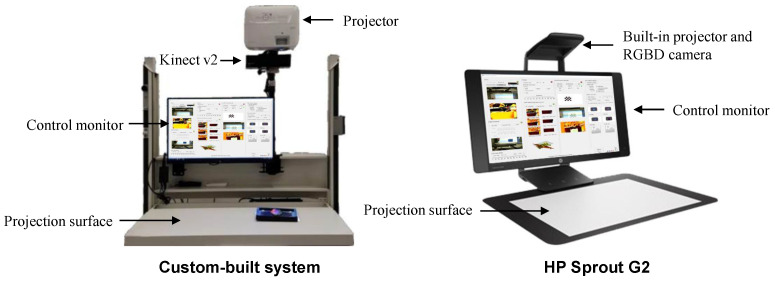
System illustration of custom-built PPM system on the left and the HP Sprout Pro G2 multimedia system on the right.

Figure 2 illustrates our custom-built setup comprising a standard PC, an RGB-D sensor, and a single projector. The PC was equipped with an Intel(R) Xeon(R) CPU E3-1245 v5@3.50GHz, 16GB of RAM, and an NVIDIA Quadro K620 graphics card. Our choice for the RGB-D camera was the Microsoft Kinect v2, with an RGB camera with a resolution of 1920 × 1080 pixels and an infrared camera (depth camera) with a resolution of 512 × 424 pixels. To facilitate Projection Mapping, we used a BenQ TH671ST projector with a resolution of 1920 × 1080 pixels for demonstration purposes. The projector and the Kinect were fixed to an arm facing downward with a distance of 100 mm from the surface of the interset.

##### HP Sprout

For the PPM system, we also used an HP Sprout Pro G2 multimedia device [30]. This device consists of a built-in PC (Intel Core i7-7700T, 16 GB DDR4 memory, NVIDIA GeForce GTX 960M), a high-resolution DLP projector (1920 × 1280), an HP high-resolution downward-facing camera (4416 × 3312), a downward-facing RGB-D camera (Orbbec Astra S Mini, RGB image resolution: 640 × 480 @30fps; depth image resolution: 640 × 480 @30fps), and an integrated 23.8″ Touch Display [31].

The software for calibration, 3D image reconstruction, and interactive Projection Mapping for both setups was developed in-house.

#### 2.1.2. PPM Calibration

An interactive PPM system was designed for surface reconstruction and Projection Mapping. The RGB-D camera in the setups will provide a stream of depth images, as well as corresponding top-view RGB images. The depth images were used for 3D surface reconstruction, and the RGB frames were captured and shown to the user on the screen for the selection of Points of interest (POIs). Furthermore, The projector in the setups was used to illuminate the target surface with bright spots corresponding to the POIs selected by the user. However, for such a system, a calibration step is essential for accurate Projection Mapping. During the calibration process, models will be estimated for the correction and transformation of depth images and extracted meshes to projector coordinates.

**Figure 3:**
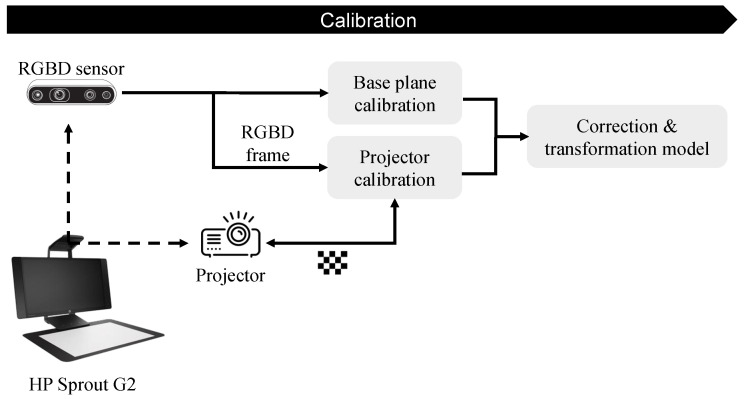
Point Projection Mapping calibration pipeline.

As demonstrated in Figure 3, the pipeline of calibration has two phases: (1) base-plane calibration and (2) projector calibration. It is worth mentioning that the calibration pipeline was identical for both setups.

#### 2.1.3. Base-Plane Calibration

**Figure 4:**
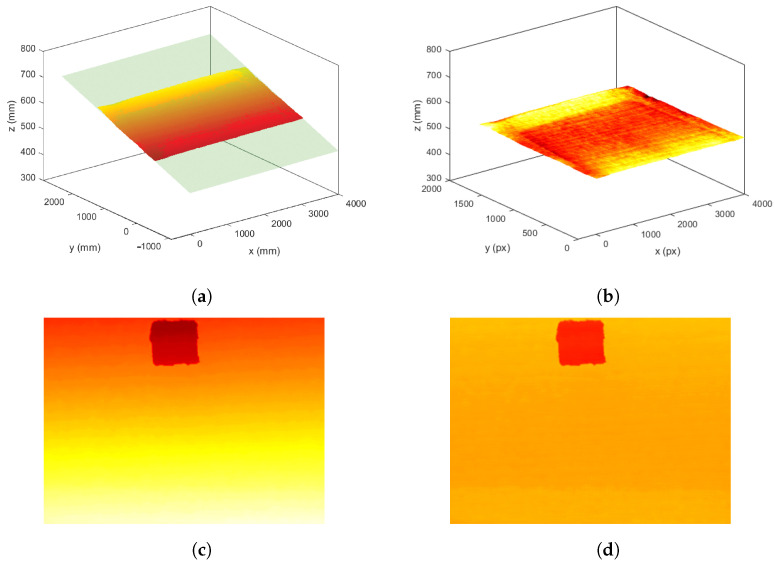
Base-plane calibration: (**a**) the 3D representation of the camera field of view flat surface before calibration where the green plane represents the plane fit to a set of randomly selected Points, (**b**) the 3D representation of the same flat surface after calibration, and (**c**,**d**) an example of a depth frame with an object before and after calibration.

The built-in RGB-D camera in the HP sprout and Kinect sensor faced downward, and in the case of having a flat surface, the depth camera should return a uniform depth image. However, the captured target surface beneath the camera was not always completely horizontally aligned with the camera’s sensor. For an accurate 3D surface reconstruction and projector calibration, a base-plane calibration step was required to discard the deviation caused by an inclined surface. For the base-plane calibration, a series of depth frames was captured and averaged to reduce any noise presence by considering the base-plane model in depth as ax+by+z+c=0 with *a*, *b*, and *c* as the base-plane model parameters. Afterward, a set of sample Points ($P_{i}\left(x_{i},y_{i},z_{i}\right)$) were randomly selected and used to compute the plane that best fit this set of Points by calculating the least squares of the normal distance to the plane, as shown in (1).

(1) $$\min\frac{1}{n}\sum_{i=1}^{n}{\left(ax_{i}+by_{i}+z_{i}+c\right)}^{2}$$

where *a*, *b*, and *c* are the parameters to minimize the least-squared error by means of partial derivatives. After obtaining the base-plane model with estimated parameters *a*, *b*, and *c*, the compensation for the deviation of the inclined surface can be performed by correcting the depth values for any Point ($P_{j}\left(x_{j},y_{j},z_{j}\right)$) in a newly captured depth frame, as shown in (2).

(2) $$z_{j}^{new}=z_{j}+ax_{j}+by_{j}+c$$

where $z_{j}^{new}$ is the corrected depth for $P_{j}$ at the spatial coordinate of ($x_{j}$,$y_{j}$). The 3D representation of the base plane before and after correction, as well as an example of the captured depth frame with an object are shown in Figure 4.

#### 2.1.4. Projector Calibration

**Figure 5:**
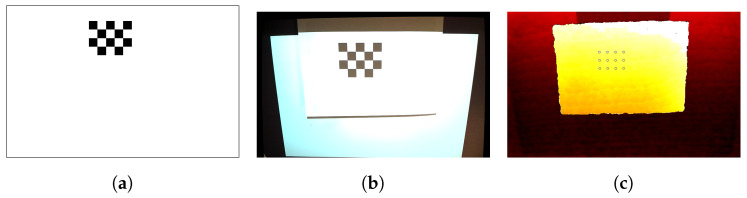
Projector calibration: (**a**) checkerboard pattern example, (**b**) corresponding acquired RGB image, and (**c**) depth image after the Projection of the checkerboard pattern.

The PPM system requires a precise transformation model to function properly. To address this need, a convenient and efficient calibration approach was deployed that was both fast and easy to execute. To implement this approach, two 3D orthogonal spaces with the Cartesian coordinate system were defined: camera space and projector space. In the camera space, an arbitrary Point is denoted by $P_{c}\left(x_{c},y_{c},z_{c}\right)$, while in the projector space, an arbitrary Point is denoted by $P_{p}\left(x_{p},y_{p},z_{p}\right)$. The transformation matrix to convert the Points between these two spaces is crucial to the Projection Mapping process, as shown in (3).

(3) $$\left(\begin{array}{cc} R & T\\ 0 & 1 \end{array}\right)\left(\begin{array}{c} x_{c}\\ y_{c}\\ z_{c}\\ 1 \end{array}\right)=\left(\begin{array}{c} x_{p}\\ y_{p}\\ z_{p}\\ 1 \end{array}\right)$$

where R denotes a 3 × 3 rotation matrix and T denotes a 3×1 translation matrix. To collect representative sample Point pairs in both the depth image and screen space for computing the transformation matrix, a 4 × 5 chessboard pattern (Figure 5a) was utilized and projected onto planes of different heights above the target surface. To recognize the sample Points in the screen space, the sequences of the chessboard pattern were used at various heights and orientations, and images were captured by the RGB-D sensor (Figure 5b,c). The recognized corner Points on the chessboard were then mapped to the depth image by the registration of the RGB to the depth images. MATLAB was used to perform the recognition extraction of 12 Point pairs per checkerboard configuration to estimate the transformation model. The transformation model was estimated by solving the estimation of the parameters using a derivative-free nonlinear solver.

### 2.2. Framework for the Validation of Optical Tissue Sensing Technologies

In this section, the developed PPM system will be implemented in a newly introduced framework for registering, labeling, and validating optical Point-based measurements with histopathology. The following framework is similar and independent of the device used since the difference in the RSME for both devices is negligible. This framework was evaluated based on a use-case study performed. For this, 30 patients who underwent breast-conserving surgery at the Netherlands Cancer Institute–Antoni van Leeuwenhoek (NKI-AVL) were included, and optical Point-based tissue measurements were performed on the excised lumpectomy specimens. In this specific use case, Diffuse Reflectance Spectroscopy (DRS) measurements were performed using an optical probe. However, this framework can be applied using any other optical Point-based technique available. This study was approved by the Institutional Review Board of NKI-AVL and registered under number IRBm20-006, which did not interfere with the standard histopathology processing and subsequent diagnostic procedures.

#### 2.2.1. Measurement Pipeline

**Figure 6:**
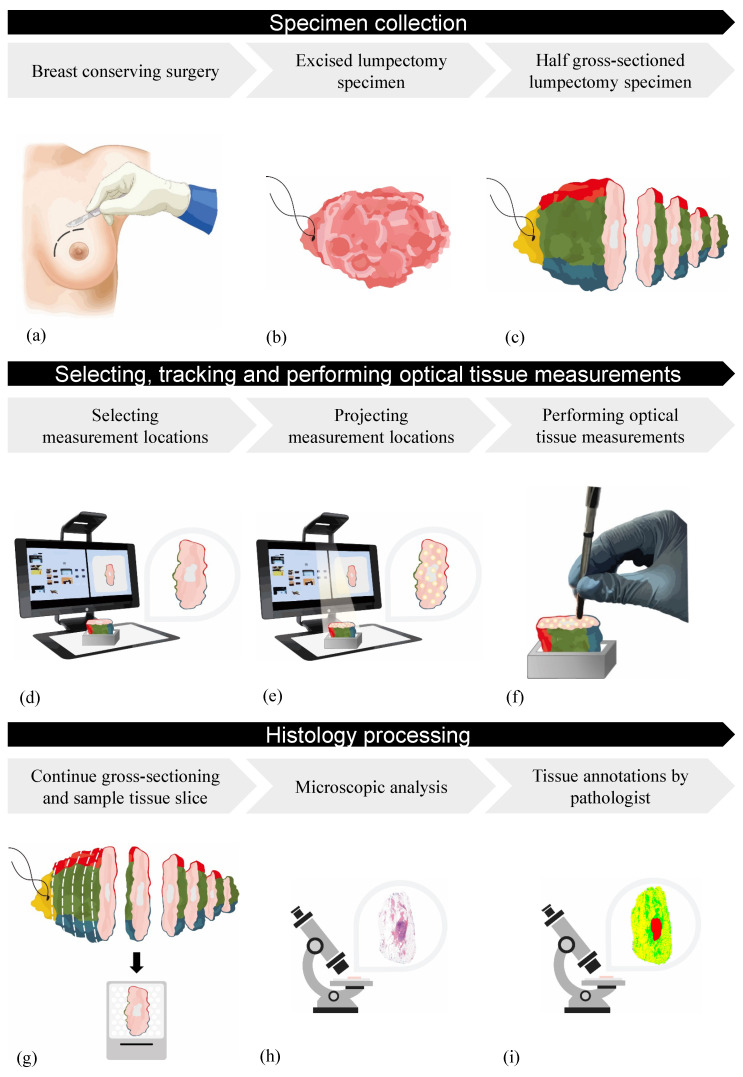
Overview of the measurement pipeline: (**a**) breast-conserving surgery, (**b**) excised lumpectomy lump, (**c**) gross-sectioning of lumpectomy lump until tumor area becomes visible, (**d**) acquiring snapshot specimen image ($S_{O}$) with PPM system and selection of measurement locations, (**e**) projecting measurement locations and acquiring a snapshot specimen including projected POIs ($S_{POI}$), (**f**) performing DRS measurements, (**g**) continued gross-sectioning and sample measured tissue slice, (**h**) processing and acquiring histology image ($H_{O}$), and (**i**) ground truth tissue annotations by pathologist and acquiring annotated histology image ($H_{A}$).

Figure 6 demonstrates the overview measurement pipeline with three main steps: (1) specimen collection, (2) selecting, tracking and performing optical measurements, and (3) histology processing.

#### 2.2.2. Specimen Collection

Immediately after the performed breast-conserving surgery, the excised lumpectomy specimen was collected in the operating theater from the NKI-AVL hospital and transported to the Department of Pathology. The specimen was inked and gross-sectioned in approximately 5 mm-thick tissue slices according to the standard protocol until either the tumor area or the placed Iodine-125 seed became visible (Figure 6a–c). The unsliced part of this lumpectomy specimen was then used for optical tissue measurements. Optical tissue measurements in this study were performed on the inside of the lumpectomy specimens, since the macroscopic appearance of tumor tissue increases the likelihood of performing measurements on tumor sites compared to optical tissue measurements that are performed on the outside of a specimen surface.

#### 2.2.3. Selecting, Tracking, and Performing Optical Tissue Measurements

The half-sliced lumpectomy specimen was positioned in a fixed holder and placed in the field of view of the PPM system. A macroscopic top-view snapshot image of the specimen was acquired and displayed on the screen (Figure 6d). From this image, Points of interest (POIs) were selected manually. The number of Points can be adjusted depending on the size of the specimen. After selection, the POIs were projected as light dots on the specimen’s surface. Next, a new macroscopic top-view snapshot image of the specimen, including projected POIs, was acquired by the PPM system (Figure 6e). The diameter of the projected POI can be adjusted to the size of the optical probe used. After these series of steps, the PPM system outputs two different specimen images: a snapshot specimen image ($S_{O}$) and a snapshot specimen image including the projected POIs ($S_{POI}$). After projecting the POIs on the specimen’s surface, optical Point-based tissue measurements were performed on each predefined location separately (Figure 6f). After positioning the probe on the POI correctly, the projector from the PPM system can be turned off so that the projected light is not interfering while performing optical tissue measurements.

#### 2.2.4. H&E Processing

Next, the remaining half-sliced lumpectomy specimen was further processed by the Department of Pathology, where sagittal slicing and gross sectioning of the lumpectomy specimen continued. The measured tissue slice, the surface of which the optical tissue measurements were performed, was then placed in a megacasette (Figure 6g). According to the standard protocol, a microscopic H&E section was created and digitalized with Aperio^®^ ScanScope AT2 (Leica Biosystems, Wetzlar, Germany) (Figure 6h). All histology images were uploaded to Slide Score (web viewer for high-resolution scans of microscopic histopathology slides). Here, for each microscopic H&E image, invasive carcinoma, ductal carcinoma in situ (DCIS), and connective and fat tissue were annotated by a pathologist and considered as the ground truth (Figure 6i). After finalizing the complete histopathology processing of the lumpectomy specimen, two different microscopic images were generated: a histology image of the measured breast specimen ($H_{O}$) and an annotated histology image of the measured breast specimen ($H_{A}$).

#### 2.2.5. Correlation with Histopathology

To summarize, after completing the measurement pipeline, four different images were obtained: two snapshot specimen images ($S_{O}$ and $S_{POI}$) and two histology images ($H_{O}$ and $H_{A}$). These images will be used in the following registration pipeline to correlate the snapshot specimen image (including the POIs) with histopathology. The histology image (including annotations of the pathologist) was used to label each optical tissue measurement with the correct pathology label.

#### 2.2.6. Automatic Deformable Image Registration

**Figure 7:**
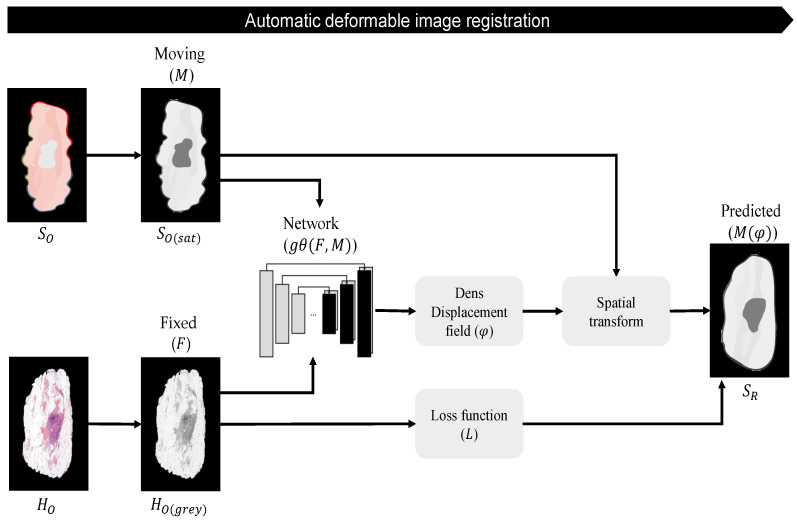
Automatic deformable image registration: $H_{O}$ is converted to a single grayscale image ($H_{O(grey)}$). For greater similarity of the intensity levels, $S_{O}$ is converted to grayscale by using the saturation values only ($S_{O(sat)}$). These images are used as the input for the unsupervised deep convolutional neural network ($g_{\theta }\left(F,M\right)$) with fixed histology image $H_{O(grey)}$ (F) and a moving snapshot specimen image $S_{O(sat)}$ (M). Mutual information is used as the loss function (L). The network outputs a dense displacement field (DDF(φ)), which defines the Mapping from the moving image coordinates to the fixed image and is used to register *M* with *F*. This results in predicted image $S_{R}(M\left(\varphi \right)$).

In a previous study, an unsupervised deep-learning-based deformable multi-modal image-registration method was developed, which is able to account for deformations between images from different modalities [32]. The architecture of this automatic deformable image-registration method is based on the VoxelMorph principle and uses a deep convolutional neural network ($g_{\theta }\left(F,M\right)$), similar to UNet [33,34], as displayed in Figure 7. The model uses two input images, in this case a fixed microscopic histology image (F) and a moving snapshot specimen image (M), which can be switched based on one’s preferences. Since this network was trained with two-channel input images, it is required to convert $H_{O}$ and $S_{O}$ to single grayscale images. To create more-comparable intensity levels between both images, the macroscopic top-view specimen image was converted to grayscale by using the saturation values only, as shown in Figure 7. Both input images were resized to 256 by 192 pixels to reduce the computational effort of the network.

The output of the model consists of a dense displacement field (DDF). This DDF has the same size as the moving image and can be defined as a set of vectors that displays the displacement of each individual pixel of this moving image. Thus, the DDF (φ) defines the Mapping from moving image coordinates to the fixed image and was used (in combination with a spatial transform function) to register both images, which results in the predicted image (M(φ)). Mutual information was used as a loss function (L), which is a common objective function for the computation of the similarity between two images acquired in different modalities.

For all 30 lumpectomy specimens, the Dice score and mutual information were calculated between the registered and unregistered images to evaluate the performance of the automatic deformable registration model. The Dice score is a commonly used metric in image registration that measures the similarity between two binary images based on the alignment of two images. The Dice score ranges from 0 to 1, where 0 indicates no overlap and 1 indicates a complete alignment between the reference and registered image. This metric mostly evaluates the shape of an image. Since a deformable registration is applied, it is also important to evaluate the overlap of the central regions in the images. This can be achieved by calculating the mutual information (MI) between two images. The basic idea of the MI in image registration is to measure the similarity between two images by comparing the histograms of these images. The MI between two images is the amount of information that is shared between their histograms. Specifically, it measures how much the joint histogram of the two images deviates from the product of their individual histograms, thereby determining the optimal alignment of two images by finding the transformation that maximizes the mutual information between them. A high MI value indicates that the images are similar and easier to align, while a low MI value indicates that the images are dissimilar and more challenging to align.

The statistical analysis was performed using IBM SPSS statistics v27 (IBM Corp., Armonk, NY, USA). A normal distribution was assessed with the Shapiro–Wilk test. The statistical analysis for normally distributed data was performed with an unpaired *t*-test and for non-normally distributed data using a Mann–Whitney test, whereas a *p*-value ≤ 0.05 was considered statistically significant.

#### 2.2.7. Label Extraction for Tissue Classification

In order to extract tissue labels for each measurement location, it is necessary to track the measurement locations in the annotated histology image (ground truth). Therefore, the first step was to extract all measurement locations from $S_{POI}$. X- and Y-coordinates of the centers of these objects were determined and a new binary image with center Points was created. Next, the measurement areas were imitated by creating circles corresponding to the size of the used optical probe (which can be adjusted based on the probed volume). Since $S_{POI}$ has the same orientation as the input image $S_{O}$, the output DDF can be used to apply the obtained deformable registration to the snapshot specimen image including the POIs. In this case, the DDF was applied to the binary image, with the same size as $S_{POI}$, to transform the extracted measurement areas to the correct orientation. By overlaying the annotated histology image $H_{A}$ with the registered binary image (with extracted measurement locations), the optically measured tissue types are visualized for each measurement location microscopically and can be considered as the ground truth. The last step involves the process of creating labels by calculating tissue type percentages for every tracked and registered measurement location. In this study, we choose a microscopic histology image as the fixed image (F) since it is easier to apply a DDF on measurement locations compared to a microscopic structure when extracting tissue labels. However, this order can be change to one’s preferences.

## 3. Results

### 3.1. Evaluation of PPM System

The accuracy of the PPM system is calculated after the calibration procedure. The root-mean-squared error (RMSE) of the transformation model was estimated by the difference between the sampling Points and the Mapping results using a checkerboard. The overall system error of the custom-built Kinect-projector setup was 0.59 mm. For the HP Sprout system, this resulted in an RMSE of 0.15 mm. The difference in the error can be due to differences in both the depth camera resolution and device stability. In the case of the HP Sprout, the projector and RGB-D camera are integrated and fixed in place, providing greater stability. However, in the custom-built system, while the projector and sensor are also fixed, they are still vulnerable to slight movements, which may impact the calibration, which could result in lower accuracy.

### 3.2. Acquired Images and Input Images

**Figure 8:**
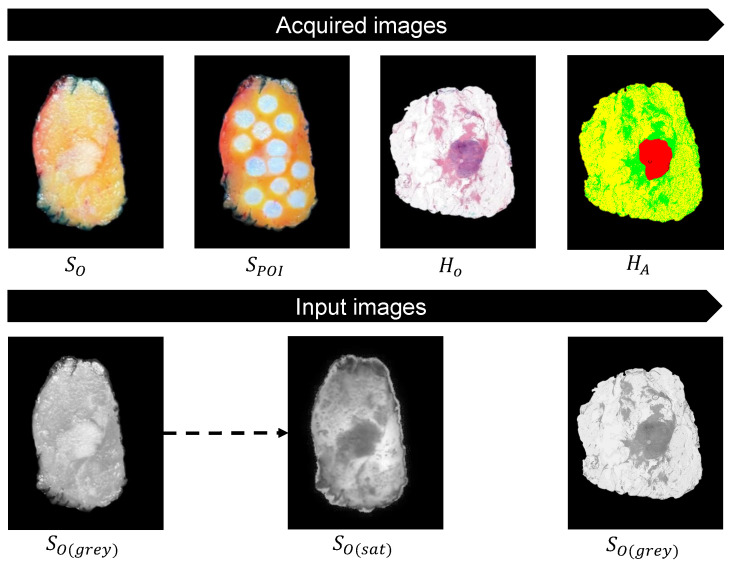
Example of the acquired images: the macroscopic top-view snapshot image of the lumpectomy specimen with and without projected POIs ($S_{O}$ and $S_{POI}$) and the microscopic histology image with and without annotations ($H_{O}$ and $H_{A}$). Both input images $H_{O}$ and $S_{O}$ were converted to single grayscale images ($H_{O(grey)}$ and $S_{O(grey)}$). For the greater similarity of the intensity levels, $S_{O(grey)}$ is converted to saturation values only ($S_{O(sat)}$).

Optical tissue measurements were obtained from 30 lumpectomy specimens, for which we completed the whole pipeline, as described in Section 2.2.1. This resulted in four different images for each specimen: $S_{O}$, $S_{POI}$, $H_{O}$, and $H_{A}$. Before using the automatic deformable image registration, the input images $S_{O}$ and the microscopic histology image $H_{O}$ were converted to grayscale. By using only saturation values, $S_{O}$ obtained similar intensity levels as $S_{O}$ (Figure 8).

### 3.3. Automatic Deformable Image Registration

**Figure 9:**
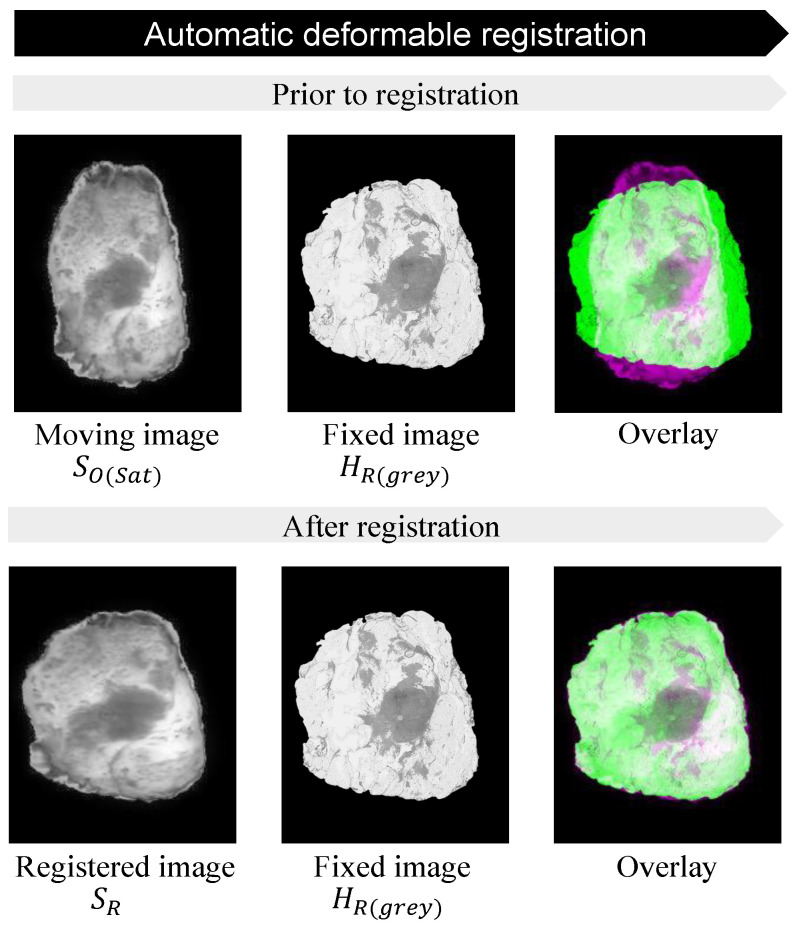
Performance of automatic deformable registration. Prior to registration: moving image $S_{O(sat)}$ (purple) laid overthe fixed image $H_{O(grey)}$ (green). After registration: predicted image $S_{R}$ (purple) laid overfixed image $H_{O(grey)}$ (green).

**Figure 10:**
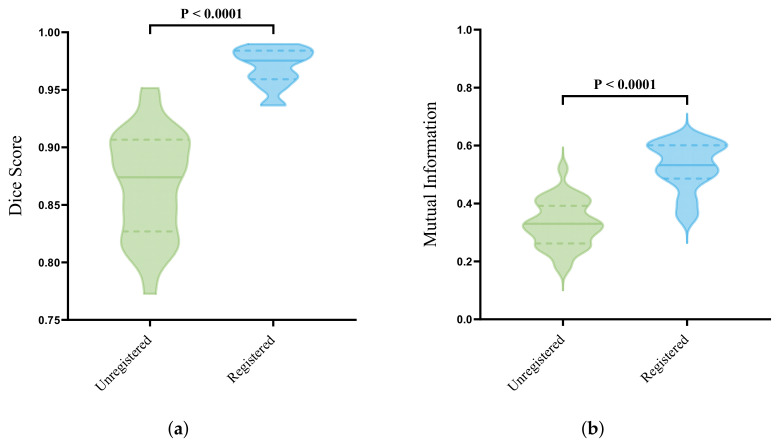
Evaluation of automatic deformable image-registration method (**a**) Dice score and (**b**) mutual information. Green and blue visualize the distribution of the unregistered and registered dataset, respectively. The middle line represents the median, whereas the thinner dotted lines represent the interquartile range (IQR).

Figure 9 shows an example for the overlap between the input images, before and after the automatic deformable image registration was applied. The results for both the Dice score and MI are visualized in Figure 10.

The violin plots show the distribution for all 30 lumpectomy specimens, before and after the registration was applied. The width of these plots shows the relative frequency in which each value occurs and becomes wider when the value occurs more frequently and with a higher probability. The distribution for the unregistered Dice score images ranges from 0.77–0.95 (median 0.86 ± 0.05) and 0.94–0.99 (median 0.97 ± 0.02) after registration was applied, whereas the distribution for the mutual information images ranges from 0.17–0.52 (median 0.33 ± 0.08) and 0.34–0.63 (median 0.52 ± 0.08) for the unregistered and registered images, respectively.

### 3.4. Label Extraction for Tissue Classification

**Figure 11:**
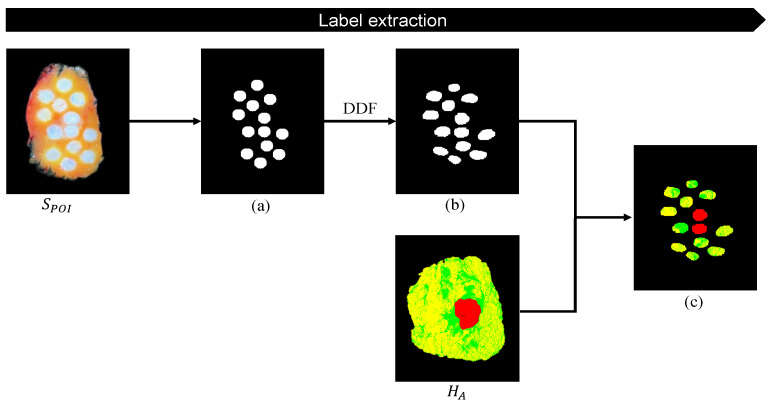
Pipeline label extraction. (**a**) Binary image of extracted measurement locations. $S_{POI}$ has the same orientation as input image $S_{O(sat)}$, so the DDF can be applied on the binary image, which results in a registered binary image with extracted measurement areas (**b**). The annotated histology image ($HE_{A}$) (where yellow, green, and red represent fat, connective tissue, and invasive carcinoma, respectively) has the same orientation as the input image $H_{O}$. Therefore, $H_{A}$ can be laid overthe registered binary image with the extracted measurement areas, resulting in the tissue label percentages used as the ground truth (**c**).

The specimen image with the projected POIs ($S_{POI}$) has the same orientation as the input image $S_{O(sat)}$. Thus, the output DDF can be applied to a binary image with extracted measurement locations to register all locations with histopathology. Therefore, the registered binary image with extracted measurement areas was laid over the annotated histology image ($H_{A}$) in order to determine the tissue labels’ percentages used as the ground truth. All steps of the framework for label extraction are visualized in Figure 11.

## 4. Discussion

The validation of optical tissue-sensing techniques is necessary before these technologies can be implemented in diagnostic tools and provide real-time tissue classification during surgical procedures. To make the performance of classification algorithms as accurate as possible, a precise method for tracking the performed optical measurements on tissue specimens is crucial. Such a method should enable measurement areas to be traced back in microscopic tissue sections and may serve as the ground truth tissue labels. However, due to histopathology processing, accurate correlation between optical tissue measurements with microscopic tissue sections is often hampered by tissue deformation. In this study, a newly developed framework was introduced for improved tracking, registering, and labeling of optical tissue measurements, which provides further validation for their clinical applicability. With the use of a Point Projection Mapping (PPM) system, the Projection of measurement locations on the tissue specimen becomes possible. The acquired top-view specimen images ($S_{O}$, $S_{POI}$) were used for the following correlation with histopathology. Using an unsupervised automatic deformable multi-modal image-registration method, measurement locations can be traced back in the annotated histology images ($H_{A}$). Labels were created by calculating the percentages of the involved tissue types for each tracked and registered measurement location.

A registration between the tracked optical tissue measurement locations and histopathology is needed to create ground truth tissue labels. Therefore, in this case study, an automatic deformable registration was applied on a newly acquired dataset of optical tissue measurements of 30 lumpectomy specimens to assess the registration performance. The distributions of the obtained Dice score and MI for the registered images were significantly higher compared to the ones obtained from the unregistered images (Figure 10). For the Dice score, the majority of the images after the registration were distributed with a median of 0.96 ± 0.01, as visualized in Figure 10a, meaning that, based on the general shape of the images, an accurate overlap was achieved. The MI was used to quantify the similarity between the different image modalities and was calculated using the histograms of the images and the joint probability distribution of their intensity values. The majority of the unregistered images were distributed around the median of 0.33 ± 0.07, whereas the majority of the cases were located above the median of 0.52 ± 0.08 after registration, meaning an improved alignment of the inside structures was achieved, as visualized in Figure 10b. The MI was originally used for comparing single modality images. However, in this study, we were dealing with registration between different image modalities with different gray intensity distributions. Although the MI gives the impression of an improvement in overlaying structures (registration), it is not the most-optimal metric to access the registration performance between multi-modal images.

The first step in the validation of technologies for optical tissue sensing involves the tracking of measurement locations. The developed PPM system showed a very high precision when projecting measurement locations on the lumpectomy specimens (RMSE of 0.15 mm using HP Sprout device) and, thereby, demonstrates added value for implementation in the proposed validation framework. It is important to note that we also utilized a custom-built device in our experiments, which yielded slightly lower, but similar performance (RMSE of 0.59). This custom-built device can be readily reproduced using any RGB-D camera and projector, addressing the concerns about the limited availability of the HP Sprout Pro G2 multimedia device. The differences in accuracy between these two systems emphasize the importance of factoring in both depth camera resolution and the sturdiness of the integration of RGB-D camera and projector when building such a PPM system.

To the best of our knowledge, this is the first automated tracking system using Projection Mapping, which minimizes tracking errors compared to other methods, for example the use of ink to mark the measurement locations. The accuracy of the ink placement can involve human error since the locations will be marked after the measurements are performed [15,16,23,35,36]. Placing ink marks prior to the measurements is not feasible, since the ink can be observed in the spectral data. Besides, the placed ink marks can diffuse to the surrounding region, resulting in the mark not exactly representing the exact measurement location. This issue also limits the number of measurements at possible Points of interest, since ink marks with the same color are not distinguishable. When measurements are performed too close together, the ink marks will overlap, which makes it even impossible to track the separate measurement locations back in the corresponding histology image. Besides, this approach is not applicable for optical measurements performed on gross-sectioned tissue slices, since the placed ink will fade during the following histopathology processes. In this case, the use of permanent fiducial markers (for example, small burn marks on the tissue slice) could be another solution to track optical tissue measurements [26,37]. However, burn marks or other permanent markers can destroy the measured tissue, and this can interfere with the following histopathology analysis, making this technique restricted to single Points of interest as well.

Using probe-fitting grids or molds is another way to track the optical tissue measurements locations without damaging the tissue [29]. But, the predefined grid locations can be insufficient since they will not always overlap with the measurement location that is aimed at. Another method to localize measurement locations is the video tracking of an optical probe [24,25]. Gorpas et al. proposed a live tracking technique for FLIm measurements by the incorporation of an aiming beam, which allows localization during acquisition. A camera acquires the locations in a white light image, from which further optical analysis is feasible [38,39]. The wavelength range used of this aiming beam does not affect the FLIm acquisition. This technique is hard to incorporate for optical techniques where the probe needs to be in contact with the tissue. Also, since this tracking method works with the use of an emitted blue light, broad-band Spectroscopy such as DRS at certain wavelengths can be affected. Blocking the field of view of a camera can also result in failed tracking, which complicates in vivo applications. In this paper, an improved method for tracking, registering, labeling, and validating optical tissue measurements with histopathology was demonstrated. With the developed PPM system, it becomes possible to project any desirable number of measurement locations in a more-controlled and -automated manner without damaging or marking the specimen. This way, human error is reduced, making this method more applicable compared to other tracking techniques available.

For this case study, lumpectomy specimens were processed in megacassettes to create microscopic histology images of the complete tissue slices. It would be desirable to apply this framework not only on lumpectomy specimens, but also within other oncology domains in which optical tissue-sensing technologies are investigated frequently and precision in correlation with histopathology is of great importance. However, when applying this framework to different types of tissue specimens, for example colon or prostate, most often, the tissue slices must be subdivided into multiple cases since the tissue specimens are too big to process in a single case or hospitals have restrictions in adjusting standard histopathology processing protocols. In that case, microscopic histology images need to be reattached before using this framework, which can be complicated by tissue deformations. Before using this framework under those conditions, small adjustments to the methodology need to be taken into consideration to process the tissue specimens and apply this framework in the most-suitable way. The Projection of POIs by the PPM system, due to base-plane and projector calibration, achieved high precision. However, the extraction of accurate tissue labels is dependent on the performance of the complete framework and relies also on the amount of tissue deformation that occurs during the histopathology processing of the tissue slices. The developed automatic deformable registration is able to accurately register borders and inside structures when registering snapshot specimen images to histology images. However, when the tissue is deformed to a certain degree, the registration and following extraction of the tissue labels will be affected. Tears, the loss of tissue, and holes make it difficult for the model to identify identical features to precisely overlay the images. This drawback is based on processes that are not related to this proposed framework, but do have an effect on the performance and need to be taken into consideration when using the obtained tissue labels for the further development of tissue classification algorithms.

The performance of the automatic deformable registration was evaluated with the use of the MI and Dice score, which determine differences in the intensity level and the overlap between the input images. We concluded that these matrices were the most-suitable to determine the registration accuracy between images in which it is difficult to find corresponding landmarks. However, other metrics such as target registration error can be explored to draw a more-definite conclusion about the performance of the model.

We would like to address that, for the validation of optical tissue-sensing techniques and their further applicability in diagnostic tools, it is of great importance to correctly label the optically measured tissue with a ground truth. By using the proposed framework, manual and time-consuming tasks will be eliminated, which results in the faster development of more-robust and -accurate classification algorithms. Once tissue classifications are developed, there is no necessity for the use of this framework, and optical techniques can be performed in real-time without utilizing the PPM system.

## 5. Conclusions

This research emphasized the critical necessity of the accurate validation of recently developed optical tissue-sensing techniques used for tissue discrimination during oncological surgery. A precise correlation of optical measurements with histological results is identified as crucial, not only for accurate validation, but also for precise labeling of optical data necessary in the development of high-performance machine learning tissue-classification algorithms.

The introduction of the Point Projection Mapping system marks a notable advancement, enabling the non-destructive tracking of measurement locations on tissue specimens. Furthermore, our proposed framework for accurate registration, validation, and labeling of optical data with histopathology results was successfully validated through a case study. The demonstrated effectiveness of the PPM system in combination with the proposed framework implies a significant step forward compared to conventional tracking techniques available. Importantly, this advancement leads to substantial time and resource savings, establishing its practicality and efficiency in validating optical tissue-sensing technologies.

## Data Availability

The data and software underlying the results presented in this paper are not publicly available at this time, but may be obtained from the authors upon reasonable request.
